# Safety, Immunogenicity, and Protective Efficacy of an H5N1 Chimeric Cold-Adapted Attenuated Virus Vaccine in a Mouse Model

**DOI:** 10.3390/v13122420

**Published:** 2021-12-03

**Authors:** Weiyang Sun, Zhenfei Wang, Yue Sun, Dongxu Li, Menghan Zhu, Menglin Zhao, Yutian Wang, Jiaqi Xu, Yunyi Kong, Yuanguo Li, Na Feng, Tiecheng Wang, Yongkun Zhao, Songtao Yang, Yuwei Gao, Xianzhu Xia

**Affiliations:** 1 Key Laboratory of Jilin Province for Zoonosis Prevention and Control, Changchun Veterinary Research Institute, Chinese Academy of Agricultural Sciences, Changchun 130122, China; sunweiyang1987@163.com (W.S.); zhenfei0420@163.com (Z.W.); 15684055980@163.com (Y.S.); lidongxu1014@163.com (D.L.); zhumenghan818@163.com (M.Z.); xdyh-1120@163.com (M.Z.); yutyanwong@outlook.com (Y.W.); xjqsdnu@163.com (J.X.); qfkongyy@163.com (Y.K.); liyuanguo0520@163.com (Y.L.); fengna0308@126.com (N.F.); wgcha@163.com (T.W.); zhaoyongkun1976@126.com (Y.Z.); yst62041@163.com (S.Y.); 2 College of Animal Science and Technology, College of Veterinary and Medicine, Jilin Agricultural University, Changchun 130118, China; 3 Jilin Province Key Laboratory on Chemistry and Biology of Changbai Mountain Natural Drugs, School of Life Sciences, Northeast Normal University, Changchun 130024, China; 4 College of Veterinary Medicine, Shanxi Agricultural University, Jinzhong 030801, China; 5 Joint National Laboratory for Antibody Drug Engineering, School of Basic Medical Sciences, Henan University, Kaifeng 475001, China; 6 Key Laboratory of Animal Resistant Biology of Shandong, College of Life Sciences, Shandong Normal University, Jinan 250014, China; 7 Jiangsu Co-Innovation Center for Prevention and Control of Important Animal Infectious Diseases and Zoonoses, Yangzhou University, Yangzhou 225009, China

**Keywords:** H5N1 influenza virus, chimeric vaccine, mice, immune protection

## Abstract

H5N1 influenza virus is a threat to public health worldwide. The virus can cause severe morbidity and mortality in humans. We constructed an H5N1 influenza candidate virus vaccine from the A/chicken/Guizhou/1153/2016 strain that was recommended by the World Health Organization. In this study, we designed an H5N1 chimeric influenza A/B vaccine based on a cold-adapted (ca) influenza B virus B/Vienna/1/99 backbone. We modified the ectodomain of H5N1 hemagglutinin (HA) protein, while retaining the packaging signals of influenza B virus, and then rescued a chimeric cold-adapted H5N1 candidate influenza vaccine through a reverse genetic system. The chimeric H5N1 vaccine replicated well in eggs and the Madin-Darby Canine Kidney cells. It maintained a temperature-sensitive and cold-adapted phenotype. The H5N1 vaccine was attenuated in mice. Hemagglutination inhibition (HAI) antibodies, micro-neutralizing (MN) antibodies, and IgG antibodies were induced in immunized mice, and the mucosal IgA antibody responses were detected in their lung lavage fluids. The IFN-γ-secretion and IL-4-secretion by the mouse splenocytes were induced after stimulation with the specific H5N1 HA protein. The chimeric H5N1 candidate vaccine protected mice against lethal challenge with a wild-type highly pathogenic avian H5N1 influenza virus. The chimeric H5 candidate vaccine is thus a potentially safe, attenuated, and reassortment-incompetent vaccine with circulating A viruses.

## 1. Introduction

Influenza virus H5N1 is a major pathogen related to public health concerns worldwide. H5N1 infects domestic poultry, wild birds, humans, and other mammals. It is divided into highly pathogenic avian influenza (HPAI) and low pathogenic avian influenza (LPAI) according to the pathogenicity in chickens. Based on the hemagglutinin gene phylogenetic characterization and sequence homology, H5N1 evolved into clades 0–9, as defined by the World Health Organization (WHO), the Food and Agriculture Organization of the United Nations (FAO), and the World Organization for Animal Health (OIE) [1]. Some clades can be divided into 3 or 4 subclades. H5 HPAI has evolved in recent years. Since 1996 when HPAI H5N1 was first detected in Chinese geese [2], this virus has been detected in poultry and wild birds in more than 50 countries [3,4,5,6,7]. With increased HPAI outbreaks in poultry, the consecutive infection with the H5N1 virus in humans has increased. The first human infection by H5N1 was reported in China, Hong Kong Special Administrative Region, in 1997 [8]. Since then, sporadic human infections by H5N1 have been reported across Asia, Africa, Europe, and the Middle East [9,10]. As of 8 August 2021, there have been 862 confirmed human cases and 455 deaths from avian influenza H5N1 [11]. Thus, the H5N1 influenza virus remains a continuous threat to humans worldwide.

The live influenza vaccine is one of the most common influenza vaccines. The live influenza vaccine offers more advantages, such as the induction of mucosal antibodies, cell-mediated immunity, and ease of administration. IgA antibody responses play a major role in live influenza vaccine protection [12]. The licensed cold-adapted (ca) influenza vaccine is used in the USA, Russia, and Europe. The master donors of licensed live influenza vaccines are from the USA and Russia; namely, A/Leningrad/134/17/57(H2N2) ca, B/USSR/60/69 ca, A/Ann Arbor/6/60 ca, and B/Ann Arbor/1/66 ca. These four master donors have been licensed for human use. In addition, new master donor viruses have been developed from A and B influenza viruses. However, under well-controlled laboratory conditions, these vaccines have a chance to adapt to grow at high temperatures [13]. Therefore, we need to design new strategies to solve the potential problems.

There are several H5 vaccine candidates based on a master donor of influenza A virus (IAV), for example, H5N1, H5N2, H5N8, and other strains [14,15,16]. Some of these vaccines have been evaluated in clinical trials [17,18]. However, the H5-based vaccines used in most studies have been derived from a previously isolated strain. Recently, several studies have been performed on other vaccines from the influenza virus [19,20,21]. New strategies have been developed for the live influenza vaccine, including an NS1-truncated or deleted, M2-deficient single replication vaccine, and optimized antigens [22,23,24,25]. There are some reports about chimeric HA and NA genes between influenza A virus and influenza B virus [26,27,28]. The incorporated influenza assembly and the packaging signal functions have been studied in detail [29]. Humans can become infected with various influenza viruses, such as H1N1, H3N2, H5N1, H7N9, etc. The influenza A virus backbone has a possibility of reassortment among other influenza A viruses. In this study, we developed a novel chimeric influenza A/B vaccine based on previous studies. We used a cold-adapted influenza B virus backbone and modified the ectodomain of the H5N1 hemagglutinin protein.

The H5N1 influenza virus has evolved and the antigenic nature of emerging clades has circulated. The WHO coordinates influenza candidate virus vaccines for influenza pandemic preparedness every year. We designed an H5 chimeric candidate virus vaccine. This vaccine was based on a cold-adapted influenza B virus, and the B/Vienna/1/99 strain, and the polybasic cleavage was deleted from the HA gene of this H5N1 virus. The modification of the H5 HA gene was chimeric into the master donor of IBV. This chimeric H5 vaccine candidate virus was a safe, attenuated, and reassortment-incompetent virus with wild-type H5 influenza virus in nature, suggesting that it can be potentially used against an H5 influenza pandemic.

## 2. Materials and Methods

### 2.1. Cells, Proteins, and Viruses

Madin-Darby canine kidney (MDCK) cells were cultured in Dulbecco’s modified Eagle’s medium (DMEM) supplemented with 5% fetal calf serum (FCS). Human embryonic kidney cells (293T) were cultured in DMEM supplemented with 10% FCS, 2 mM glutamine, 10 mM HEPES, and 100 mg/mL streptomycin or 100 IU/mL penicillin. MDCK and 293T cells were cultured as described previously [30]. The A/chicken/Guizhou/1153/2016 (H5N1) (GenBank number: MT126478.1) virus is a candidate vaccine virus recommended by the WHO global strategy for pandemic preparedness. Purified HA of A/chicken/Guizhou/1153/2016 (H5N1) was purchased from Sino Biological company, expressed in insect cells. The B/Vienna/1/99 (abbreviated BV99) virus is a cold-adapted master donor virus for influenza B virus, made public in a patent (GenBank number: AX399742–AX399749) [31]. We rescued this master donor virus by eight-plasmid reverse genetics. The RE-12 antigen for HI assays was purchased from the Harbin National Engineering Research Centre of Veterinary Biologics Corporation in China. The H5N1 virus A/Lion/Wuhan/EZ02/2016 (abbreviated as EZ02) is a wild-type influenza virus that belongs to the same clade as the H5 virus vaccine for challenging mice and for conducting serum assays. The EZ02 virus was isolated at our laboratory in 2016 from a lion in the Ezhou zoo, in Hubei province, China. We propagated these viruses in 8–10-day-old embryonated chicken eggs, after which the allantoic fluids were collected and stored at −80 °C.

### 2.2. Vaccine Rescued and Characteristics Analysed

The eight-plasmid system of the B/Vienna/1/99 strain was constructed by a bidirectional transcription vector pBD, as previously described [32]. The H5 HA gene was modified in its polybasic cleavage site from HPAI to LPAI and synthesized by Shanghai Generay Biotechnology, China. The H5 chimeric HA gene was modified with the ectodomain of the A/chicken/Guizhou/1153/2016 virus and the signal peptide and transmembrane domain of the influenza B virus. The plasmid was constructed by using infusion technical methods (Cat. 638947, Takara). The HA gene-specific primers were used to amplify the HA ectodomain (F primer 5’-GTAACATCCAACGCAGATCATATTTGCATTGGATATCATG-3’; R primer 5’-AGTATGATTATCCAATGACTCCAATTTTACTCCAC-3’). Vector-specific primers were utilized for the cloned pBD vector with a signal peptide, transmembrane domain, and cytoplasmic domain of the IBV HA gene (F primer 5’-TTGGATAATCATACTATACTGCTCT-3’; R primer 5’-TGCGTTGGATGTTACTACCATGAGT-3’). The recombined vaccine virus was generated through classical reverse genetics. Briefly, the H5 chimeric HA plasmid and the other seven gene plasmids of MDV were co-transfected into 293T cells in 6-well plates at 33 °C under a 5% CO_2_ atmosphere. Transfections were performed with 600 ng of each plasmid with 10–15 μL of lipofectamine 3000, and the medium was replaced with Opti-MEM after 12 h. After 3 days, MDCK cells or 8-day-old embryonated chicken eggs were inoculated with the cultures and incubated at 33 °C. We used 1% chicken red blood cells to evaluate the final results. The H5 rescued vaccine virus was abbreviated as rA/B-GZ16-ca. Eight genomes of the rescued virus were sequenced to ensure the sequence authenticity by Sanger sequencing methods, as described previously [33]. The PCR amplification primers are shown in Supplementary Table S1.

The temperature sensitivity and cold-adapted phenotype were determined by 50% egg infective dose (EID_50_) titers at 27, 33, and 39 °C. A temperature-sensitive phenotype was defined by virus growth at 33 °C, while restricted growth was defined as that at 39 °C. If a virus titer at 39 °C was lower by three log_10_ EID_50_ compared with 33 °C, the virus was considered to be temperature-sensitive. The cold-adapted phenotype was determined by titration of the virus with eggs at 33 and 27 °C. The viruses were considered cold-adapted if the titer was no more than three log_10_ EID_50_ lower at 27 °C when compared to that at 33 °C. The reproduction capacity at restrictive temperature (RCT) was defined as RCT_39_ [log_10_(EID_50_ mL^−1^) = [log_10_(EID_50_ mL^−1^)] at 33 °C- [log_10_(EID_50_ mL^−1^)] at 39 °C, and RCT_27_ [log_10_(EID_50_ mL^−1^)] = [log_10_(EID_50_ mL^−1^)] at 33 °C- [log_10_(EID_50_ mL^−1^)] at 27 °C-.

### 2.3. Viral Growth Kinetics in Cell Culture

We evaluated the growth kinetics of the H5 vaccine virus in MDCK cells. Twelve-well plates were used to culture the cells infected with the H5 vaccine virus at a multiplicity of infection (MOI) of 0.01. Fifty percent of tissue culture infectious doses (TCID_50_) were used per cell. The cells were cultured in DMEM containing 2 μg/mL of TPCK-treated trypsin, and the cells were incubated at 37 °C under a 5% CO_2_ atmosphere. At 12, 24, 36, 48, 60, and 72 h, the cells and supernatants were collected, and the virus titers were assayed in MDCK cells. The virus titration was measured by TCID_50_. All experiments were performed in triplicate.

### 2.4. Virus Titrations in MDCK Cells and Tissues

The influenza virus used in this study was titrated with MDCK cells and embryonated chicken eggs. The tissues were collected and homogenized with the TissueLyser (QIAGEN) in 1 mL of DMEM, followed by centrifugation for 10 min at 5000 rpm. We also used a 10-fold dilution series with DMEM in a volume of 0.1 mL and inoculated 8–10-day-old embryonated chicken eggs at 37 °C for 48 h with influenza A virus or at 33 °C for 48–72 h with influenza B virus. The allantoic fluids were harvested. The hemagglutinin assays were performed with 0.5% chicken erythrocytes. The virus titer was calculated according to the Reed and Muench method [34].

For end-point viral titration with MDCK cells, the monolayers of cells were inoculated in 96-well culture plates with Opti-MEM and TPCK-treated trypsin. The virus samples were diluted by 10-fold dilutions and incubated at 37 or 33 °C under a 5% CO_2_ atmosphere for 1 h. After inoculation, the plates were supplied with Opti-MEM and incubated with 5% CO_2_ for 2–3 days. The HA assays were tested with 0.5% chicken erythrocytes. The virus titer was calculated according to the Reed and Muench method described earlier.

### 2.5. Mouse Immunization, Safety, and Protection

Specific-pathogen-free (SPF) 4–6-week-old BALB/c female mice were purchased from the Experimental Animal Centre of Charles River. BALB/c mice were used to evaluate the pathogenicity of the H5 candidate virus vaccine. These mice were divided into an infected group and a control group, with 11 mice in each group. The mice were anaesthetized with isoflurane and inoculated intranasally with the H5 virus vaccine at a dose of 10^5^ EID_50_ in a 50 μL volume. The control group was inoculated with PBS. The lungs and nasal turbinates were collected on days 3 and 6 after inoculation. On day 3 post-inoculation (dpi), other tissues, such as the livers, spleen, kidneys, brains, hearts, and intestines, were collected. The viral titers of the tissues were determined by EID_50_. The body weights and survival were monitored for 2 weeks.

The female BALB/c mice were immunized intranasally with one dose or two doses of 10^5^ EID_50_ in a 50 μL volume, in 10 mice per group. The mice in the control group were inoculated with an equal volume of PBS. After three weeks, the sera were collected and assayed for antibodies, from five mice per group. The lung lavage fluids were collected from each group and assayed to detect IgA antibodies. The spleens from three mice from each group were collected and the isolated splenocytes were subjected to the ELISpot assay and cytokine assays by Luminex detection.

Immunized mice were challenged with a wild-type EZ02 H5N1 influenza virus. The mice were divided into a one-dose group, a two-dose group, and a control group, with eleven mice per group. After one dose or two doses of H5 chimeric vaccine, the mice were challenged intranasally with wild-type EZ02 at a dose of 10 MLD_50_ (50% mouse lethal dose) at a titer of 10^2.25^ EID_50_ in a 50 μL volume. Body weights and survival were recorded for 14 days. On days 3 and 6 post-inoculation, the lungs and nasal turbinates of three mice in each group were collected and titrated by EID_50_ methods. The lungs of each group were collected for histological lesion examination. Lungs were analyzed histologically on day 3 after the challenge. The lung tissues were fixed in 10% formalin/PBS and embedded in paraffin. The sections were then stained with hematoxylin and eosin (H&E), followed by immunohistochemistry staining (IHC). The secondary antibody of anti-Influenza A Virus Nucleoprotein antibody (AA5H) (ab20343; Abcam) was used in the IHC assays. At the end of the experiments, all surviving mice were humanely euthanized.

All live animal work was performed in accordance with guidelines from the Animal Welfare and Ethics Committee of the Changchun Veterinary Research Institute of the Chinese Academy of Agricultural Sciences (IACUC of AMMS-11-2019-002). The environment and housing facilities satisfied the National Standards of Laboratory Animal Requirements (GB 14925-2010) of China. Experiments with the wild-type H5N1 virus were conducted in a biosecurity level 3 laboratory, as approved by the Institutional Biosecurity Committee. 

### 2.6. Hemagglutination Inhibition Antibody Assay

The HAI assay was conducted as described previously [35]. Briefly, the blood samples from mice were centrifuged at 3000 rpm for 10 min, and the serum was collected and stored at −20 °C. Prior to the HAI assay, the serum was pre-treated with a receptor-destroying enzyme (RDE; Denka Seiken, Tokyo, Japan) at 37 °C for 16–18 h and then heat-inactivated at 56 °C for 30 min. The final serum dilution was 1:5. A standard commercial antigen of H5 influenza RE-12 and inactivated wild-type EZ02 were used for detecting the HAI antibodies. The test antigen was prepared for 4 HA units. The sera were 2-fold serially diluted in PBS in V-bottom, 96-well micro-plates. The diluted sera and 4 HA units were incubated at room temperature for 30 min. The HAI assay was performed with 0.5% chicken erythrocytes.

### 2.7. Micro-Neutralizing (MN) Assay

After the sera were pre-treated with RDE, as described for the HAI assays, the 1:5 diluted sera were mixed with the wild-type EZ02 at 100 TCID_50_ and incubated at 37 °C for 1 h. The samples were then incubated with the MDCK monolayer cells at 37 °C for 1 h, after which the liquid was discarded and replaced with Opti-MEM, containing streptomycin, penicillin, and 2 μg/mL of TPCK-treated trypsin. The cells were cultured at 37 °C under a 5% CO_2_ atmosphere for 2–3 days. The titers of neutralizing antibodies were defined as the highest dilution of serum that led to complete neutralization, as determined by the HA test. The HA assays were performed with 0.5% chicken erythrocytes.

### 2.8. Enzyme-Linked Immunosorbent Assay (ELISA)

Purified H5 vaccine HA protein was used for the ELISA assay. Microplates (high-sorbent, 96-well plate) were coated with 100 μL/well of 5 μg/mL H5 vaccine HA protein at 4 °C overnight. The coated plates were washed with PBS-T and incubated with 1% bovine serum albumin in PBS at 37 °C for 1 h. The serum was diluted from 1:20 for IgG antibodies, and the lung lavage fluids were diluted from 1:10 for IgA antibodies. The plates were washed with PBS-T 3 times, with a 150 μL volume for each well. After washing, the plates were incubated with goat anti-mouse IgG H&L (ab6789; Abcam) at a dilution of 1:100,000 or goat anti-mouse IgA H&L (ab97235; Abcam) at a dilution of 1:100,000. The plates were washed with PBS-T, and the tetramethylbenzidine (TMB) substrate solution was added for 30 min, after which the reaction was terminated with the addition of a stopping solution. The optical density was measured at 450 nm with a plate reader (Bio-Rad, Hercules, CA, USA). The wells were considered positive when the OD_450_ value was at least two-fold more remarkable than that of control wells. The IgA and IgG antibody titers were accessed by the highest dilution with OD_450_.

### 2.9. Protein Microarray Assay

A protein microarray was performed to detect the IgG and IgA antibodies in the mouse sera. Thirty-nine H5 influenza HA proteins (Supplementary Figure S1) belonging to different clades were purchased from Sino Biological and Cambridge Biologics. The antigens belong to Clade 0, Clade 1, Clade 2.2, Clade 2.3.4, Clade 2.3.2.1, Clade 2.3.4.4, and other clades. The HA antigens were reconstituted to a 10 μg/mL concentration with phosphate-buffered saline (PBS) and then printed on the microarray slides. The sera were diluted 1:300 with the protein microarray buffer. In brief, the arrays were incubated with a blocking buffer (PBS with 0.001% Tween-20 and 1% BSA) for 30 min, followed by washing the arrays 3 times with TBST buffer (20 mM Tris-HCl, pH 7.6, 150 mM NaCl, 0.05% Tween-20). The diluted sera were added to the arrays and incubated for 1 h at 60 rpm. The goat-anti-mouse IgG H&L (Cy5 ®), preabsorbed (ab6563; Abcam), was added at the recommended dilution and incubated for 30 min. The arrays were washed three times, air-dried, and centrifuged at 60 rpm for 10 min. The imager settings were set to 655 nm channels. The slides were imaged using an ArrayCam imager to measure the fluorescence signals.

### 2.10. ELISpot Assay

The ELISpot assay was performed to measure the expression of IFN-γ and IL-4 in immunized mice. The splenocytes were isolated from the immunized mice group and the control group. Pre-coated IFN-γ and IL-4 ELISpot plates (Mabtech AB, Stockholm, Sweden) were incubated with single-cell spleen suspensions at the concentration of 1 × 10^6^ cells/mL in 96-well plates. These cells were then stimulated with 10 μg/mL of purified HA protein from the A/chicken/Guizhou/1153/2016 (H5N1) virus. After 24–36 h of incubation at 37 °C under a 5% CO_2_ atmosphere, the amounts of secreted IFN-γ and IL-4 cytokines were measured. According to the standard protocol, these results were quantified with an ELISpot reader (Multispotreader Spectrum, AID, Strasberg, Germany).

### 2.11. Luminex Assay

Luminex technology was used to measure the number of cytokines in the supernatants of the splenocytes cultures. The splenocytes were obtained as described for the ELISpot assays. We used a 6-well plate to culture the isolated splenocytes with or without stimulation with an H5 vaccine HA protein. After incubation under a 5% CO_2_ atmosphere at 37 °C, the culture supernatants were collected and stored at −80 °C. The supernatants were measured using the Th1/Th2 Cytokine 11-Plex Mouse Panel kit (Cat. No. EPX110-20820-901, Invitrogen) using the Luminex 200 instrument, as described by the manufacturer instructions.

### 2.12. Statistical Analysis

GraphPad Prism 8.0 software (GraphPad Software, San Diego, CA, USA) was used to analyze the study data. The data were expressed as the mean ± standard deviation (SD) and analyzed by an unpaired t-test or Mann-Whitney test. A comparison was considered to be statistically significant when *p* < 0.05.

## 3. Results

### 3.1. Biological Characteristics of the H5N1 Influenza Vaccine

**Figure 1:**
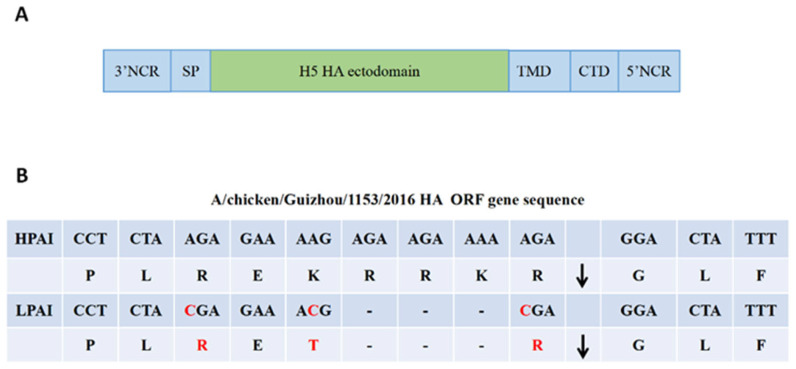
Chimeric HA design and construction. (**A**) The H5 HA ectodomain was sourced from the A/chicken/Guizhou/1153/2016 (H5N1) strain and the packaging signals, transmembrane domains, and the cytoplasmic domains were sourced from the influenza B virus. (**B**) The polybasic cleavage site in H5 HA was modified from HPAI to LPAI. 3’NCR: 3’ non-coding region; SP: signal peptide; TMD: transmembrane domain; CTD: cytoplasmic domain; 5’NCR: 5’ non-coding region.

We designed a chimeric influenza A/B virus vaccine in this study. Briefly, we transferred an H5N1 HA ectodomain to the master donor of IBV. We constructed an H5N1 candidate vaccine based on the WHO recommendation for H5 preparedness antigens. As reported previously, the signal peptide, transmembrane domain, and cytoplasmic domain were retained from HA in IBV [26]. This strategy contained the packaging signals from the influenza B virus, which helped to rescue the virus (Figure 1A). The polybasic cleavage site in HA was deleted, and the virus was modified from a high pathogenic avian virus to a low pathogenic avian virus. The amino acids in the multi-polybasic cleavage site were changed from PLREKRRKRGLF to PLRETRGLF (Figure 1B). Then, the modified H5 HA gene and the other 7 master donor plasmids from the influenza B virus were co-cultured with 293T cells and MDCK cells using reverse genetic methods. The chimeric A/B virus vaccine was rescued successfully and named rA/B-GZ16-ca. Sanger sequencing confirmed the expected viral genome.

We evaluated the temperature sensitivity and cold-adapted phenotypes as well as the growth of the virus in 8–10-day-old embryonated chicken eggs. The master donor of influenza B virus had a titer of 7.7 ± 0.5 log_10_ EID_50_/mL at 33 °C and a titer of 6.2 ± 0.2 log_10_ EID_50_/mL at 27 °C (Table 1). The results revealed that the MDV of the influenza B virus was temperature-sensitive and cold-adapted. Moreover, the rA/B-GZ16-ca virus had a titer of 8.2 ± 0.2 log_10_ EID_50_/mL at 33 °C and reached a titer of 6.9 ± 0.7 log_10_ EID_50_/mL at 27 °C (Table 1). The virus could not grow in 8–10-day-old embryonated chicken eggs at 39 °C. The virus titer at 27 °C was no more than 1000 times that of the titer at 33 °C. The virus titer at 33 °C was greater than 1000 times the titer at 39 °C. Thus, the results indicate that the chimeric virus was temperature-sensitive and cold-adapted.

**Figure 2:**
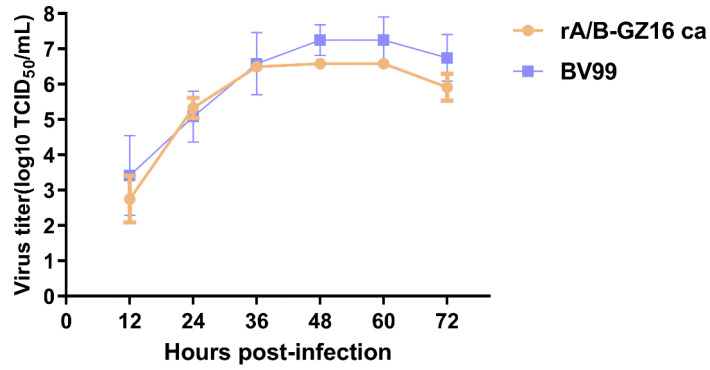
Growth of MDV and H5 virus vaccine in MDCK cells. MDCK cells were inoculated at a multiplicity of infection of 0.01. The supernatants were collected at 12, 24, 36, 48, 60, and 72 h. The virus titer was measured with MDCK cells by the TCID_50_. The titers are presented as the mean log_10_ TCID_50_/mL ± SD.

We also evaluated the growth of the H5 virus vaccine and the master donor of the influenza B virus. The rA/B-GZ16-ca virus was also titrated with MDCK cells. The virus reached a high titer at 48 h post-infection (Figure 2).

### 3.2. Safety of H5 Virus Vaccine

**Figure 3:**
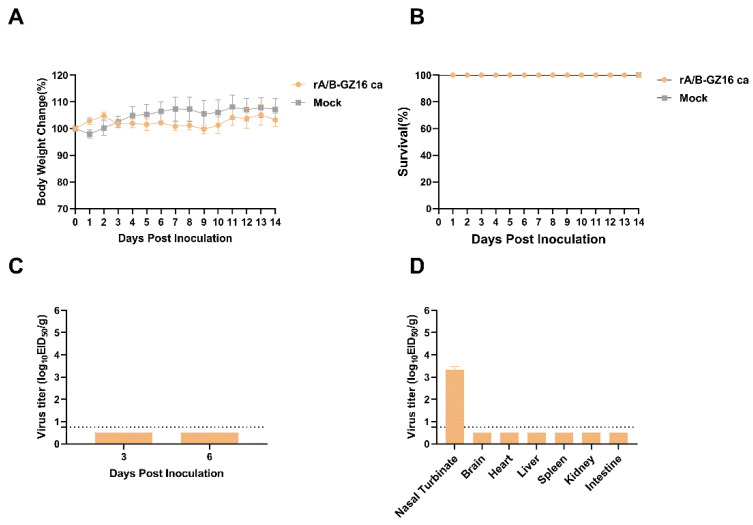
Safety evaluation of H5 virus vaccine in mice. The mice were divided into the infected group and the control group (*n* = 11). The lungs, nasal turbinates, and other tissues from the mice were collected as described in the Methods Section. (**A**) Body weight at 14 days after infection (*n* = 5). (**B**) Survival during the 14 days (*n* = 5). (**C**) Virus titer of the lungs on days 3 and 6 post-infection (*n* = 3). (**D**) Nasal turbinates and other tissues on day 3 (*n* = 3). Data are shown as the mean ± SD. The limit of detection was set to 0.75 log_10_ EID_50_/g.

We infected BALB/c mice with the H5 candidate virus vaccine at a dose of 10^5^ EID_50_ in 50 μL. Mice in the control group did not show body weight loss, and none of them died (Figure 3A,B). The infected group of mice survived to 14 days, and none of them died. The infected mice did not experience body weight loss. The virus had not replicated in the lungs of infected mice by days 3 and 6 post-infection (Figure 3C). On day 3, we detected low virus replication in nasal turbinates—the titer was 3.5 log_10_ EID_50_/g (Figure 3D). The virus was not detected in the brains, livers, spleens, kidneys, or intestines. In summary, the results revealed that our H5 candidate vaccine was attenuated and safe in the experimental mice.

### 3.3. Antibody Immune Responses in Mice

#### 3.3.1. Humoral Immune Responses

To evaluate the antibody immune responses, the mice were immunized with the H5 virus vaccine with one dose or two doses of 10^5^ EID_50_ in a 50 μL volume. After 3 weeks, the sera were used to measure the antibody immune responses. Since we did not have the wild-type A/chicken/Guizhou/1153/2016 strain, we instead used the wild-type EZ02 strain from the same clade as the A/chicken/Guizhou/1153/2016 strain.

**Figure 4:**
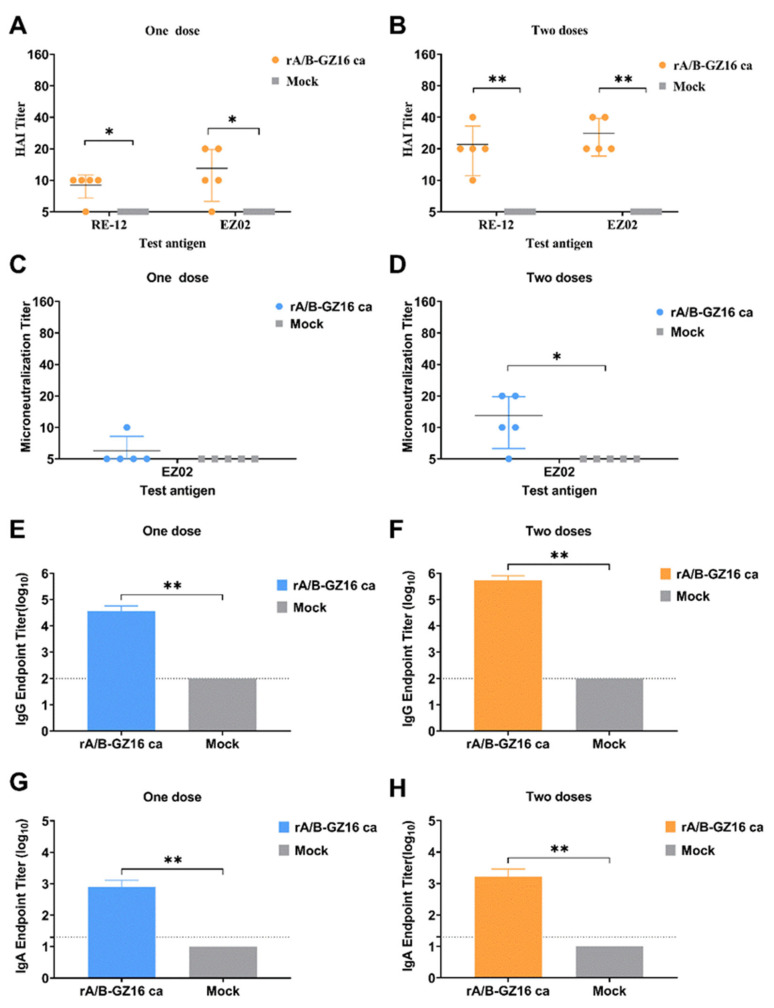
Antibodies were detected in the sera and the lung lavage fluids of immunized mice. The serum samples were collected at 3 weeks after immunization with one dose or two doses. The RE-12 and EZ02 were used as test antigens for the HAI assays, and the wild-type EZ02 virus was used as the test antigen for the microneutralization assays. The HA protein of the H5 virus vaccine was used for ELISA. The IgG antibody responses were measured in serum, and the IgA antibody responses in the lung lavage fluids were determined. (**A**) One-dose group and (**B**) two-dose group HAI assay results in immunized mice, *n* = 5. (**C**) One-dose group and (**D**) two-dose group microneutralization assay results for the immunized mice, *n* = 5. (**E**) One-dose group and (**F**) two-dose group IgG ELISA titers in mice, *n* = 5. (**G**) One-dose group and (**H**) two-dose group IgA ELISA titers measured in mice, *n* = 5. Data were analyzed by an unpaired t-test or Mann-Whitney test. Data are shown as the mean ± SD. * *p* < 0.05, and ** *p* < 0.01.

We measured the HAI titers in the sera of the immunized mice. Using RE-12 standard test antigen, we determined that the HAI antibodies of the sera in the one-dose group were lower than those in the two-dose group (Figure 4A,B). The HAI antibodies reached 1:40 in two-dose-immunized mice serum. When using EZ02 as the test antigen, we found that the HAI antibodies reached 1:20 in the one-dose group and 1:40 in the two-dose group. The results suggested that the two-dose-immunized mice produced a greater amount of HAI antibodies when compared with one-dose-immunized mice.

We performed a microneutralization assay with the wild-type EZ02 virus, which belongs to H5 HPAI. The sera from one-dose mice had a low titer, reaching 1:10. The microneutralization titers in the sera of two-dose mice were 1:20 (Figure 4C,D). These results indicated that wild-type H5N1 HPAI could induce a moderate microneutralization titer in the mice sera.

We treated sera with RDE and assayed them for IgG antibodies. Compared with the control group, the serum in the one-dose group or two-dose group contained a great amount of ELISA-detected IgG antibodies against H5 vaccine proteins, and the amount of IgG antibody was greater in the two-dose group when compared with that in the one-dose group (Figure 4E,F).

In summary, the results of the HAI assay, MN assay, and ELISA assays indicated that the H5 vaccine could produce a humoral immune response in the serum.

#### 3.3.2. Mucosal Immune Responses

Mucosal immunity offers the advantage of immune responses in live influenza vaccines. We immunized mice intranasally in the one-dose and two-dose groups to induce nasal mucosal immunity. In the lung lavage fluids, we detected the IgA antibodies by ELISA. The H5 vaccine HA protein was used as the test antigen. We did not detect the IgA antibody in the control group. The lung lavage fluids of the two-dose group contained a greater amount of IgA than those of the one-dose group (Figure 4G,H), suggesting that the H5 chimeric vaccine induced mucosal immune protection.

#### 3.3.3. Protein Microarray Antibody Responses

**Figure 5:**
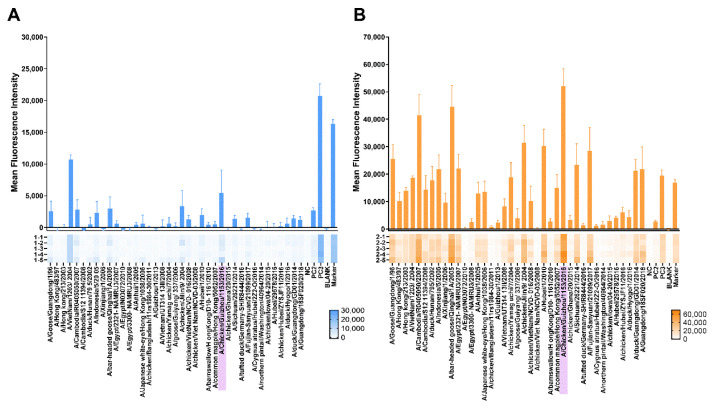
IgG antibody reactivity against H5 proteins in the serum as measured by the mean fluorescence intensity. (**A**) Protein microarrays, *n* = 5, measured the one-dose group mice serum. (**B**) The two-dose group mice serum was measured by protein microarrays, *n* = 5. The numbers listed belong to the serum. The mean fluorescence intensity (MFI) of each HA protein was determined based on the average of the median fluorescence signal of 4 replicated spots. The MFI of each HA protein was deducted from the background fluorescence signals. The higher the mean fluorescence intensity, the more antibodies bound to an HA antigen. The figures show the responses against every single HA protein antigen by the MFI on the y-axis. A/chicken/Guizhou/1153/2016 (H5N1) virus HA protein is presented in purple colour. NC: negative control; PC2: human IgG; PC3: mouse IgG; Marker: SA-Cy5.

The protein microarray method can be used to detect IgG antibodies against several types of H5 influenza HA antigens. The protein microarray results indicated that sera from the one-dose and two-dose groups had cross-reactive responses against other H5 proteins, especially the sera from the two-dose groups—the sera could have more cross-reactivity to H5 proteins from different clades (Figure 5A,B). The sera revealed high IgG serum reactivity to the A/chicken/Guizhou/1153/2016 HA antigen in the one-dose and two-dose groups. In the two-dose group, the MFI were much higher than that in the one-dose group. These results indicated that the H5N1 chimeric cold-adapted influenza vaccine may have induced a wide range of antibody reactivity against different H5 influenza viruses.

#### 3.3.4. Mice Cellular Immunity

**Figure 6:**
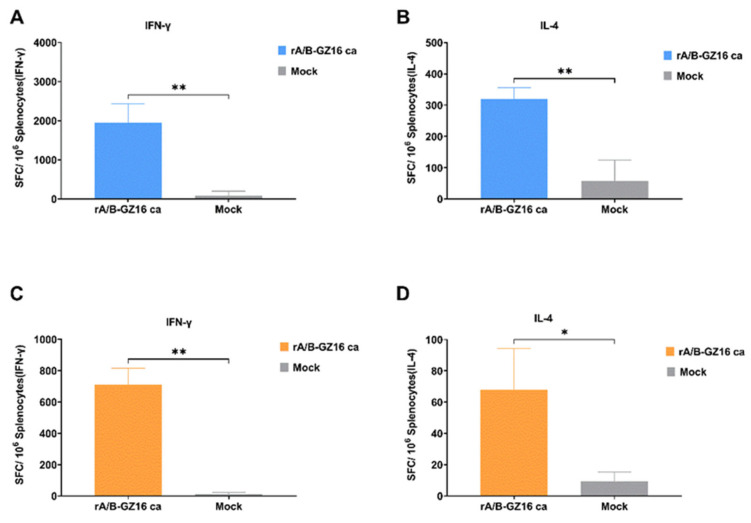
ELISpot results for splenocytes of the immunized mice. Splenocytes were collected at 3 weeks after immunization with one dose or two doses. The splenocytes were cultured and stimulated with purified HA protein from the A/chicken/Guizhou/1153/2016 (H5N1) virus, *n* = 3. (**A**) IFN-γ and (**B**) IL-4 results of the one-dose group. (**C**) IFN-γ and (**D**) IL-4 results of the two-dose group. Data are shown as the mean ± SD of the numbers of spot-forming cells (SFCs) per million splenocytes in each group. All the data were analyzed by an unpaired t-test. * *p* < 0.05, and ** *p* < 0.01.

To evaluate the cellular immunity induced by the H5 influenza vaccine, we prepared single-cell suspensions of spleens from the immunized and control mice. The cultured splenocytes were stimulated with or without purified HA proteins from the A/chicken/Guizhou/1153/2016 (H5N1) virus. We employed the ELISpot assay to measure cytokines, such as IFN-γ and IL-4, secreted by the splenocytes. Compared with the control group, the immunized mice produced significantly higher IFN-γ- and IL-4-secreted cells in the one-dose or two-dose groups (Figure 6A–D). The ELISpot results suggested that the expression of secreted IFN-γ numbers was greater than that of IL-4 in the immunized mice.

**Figure 7:**
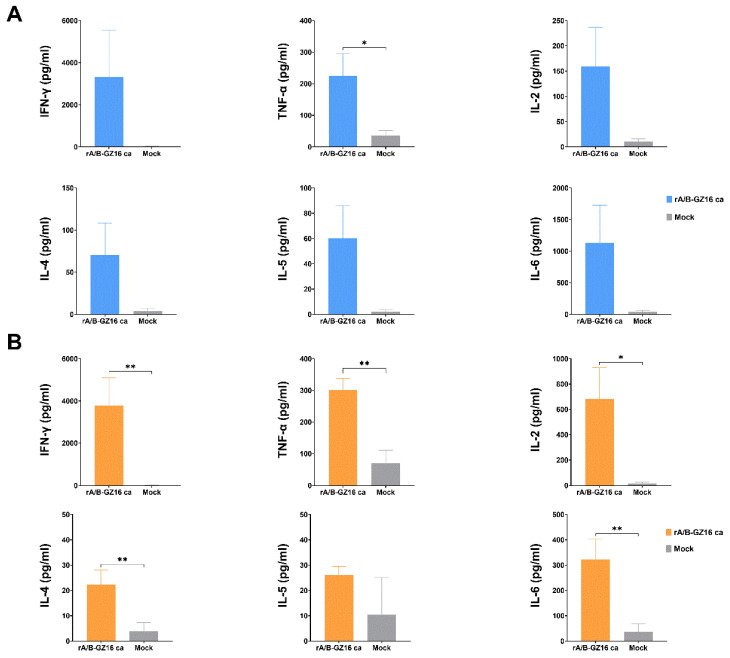
Concentrations of cytokines secreted by cultured splenocytes from immunized mice. The isolated splenocytes were stimulated with purified HA protein from the A/chicken/Guizhou/1153/ 2016 (H5N1) virus. The concentration of HA protein in each culture well was set to 10 μg/mL. The culture supernatants were collected 3 days later and subjected to the Luminex assay to measure the concentrations of multiple cytokines. (**A**) One-dose group. (**B**) Two-dose group. Data are shown as the mean ± SD. All data were analyzed by and unpaired t-test. * *p* < 0.05, and ** *p* < 0.01.

The cytokines were measured by the Luminex assay in the supernatants of splenocytes cultured in a 6-well plate and stimulated by HA proteins at the concentration of 10 μg/mL. The one-dose and two-dose groups of mice revealed an increase in the cytokine expression compared to that in the control group, including IFN-γ, TNF-α, IL-2, IL-4, IL-5, and IL-6 (Figure 7A,B). The expression of IFN-γ, TNF-α, and IL-2 was assumed to be derived from the T-helper 1 (Th1) cells. The expression of IL-4, IL-5, and IL-6 was likely derived from T-helper 2 (Th2) cells. These Th1 and Th2 cytokines have a relation to the immunity responses. Our results indicate that the H5 vaccine induced cell-mediated immunity in the immunized mice.

### 3.4. Protective Efficacy of H5 Influenza Vaccine

**Figure 8:**
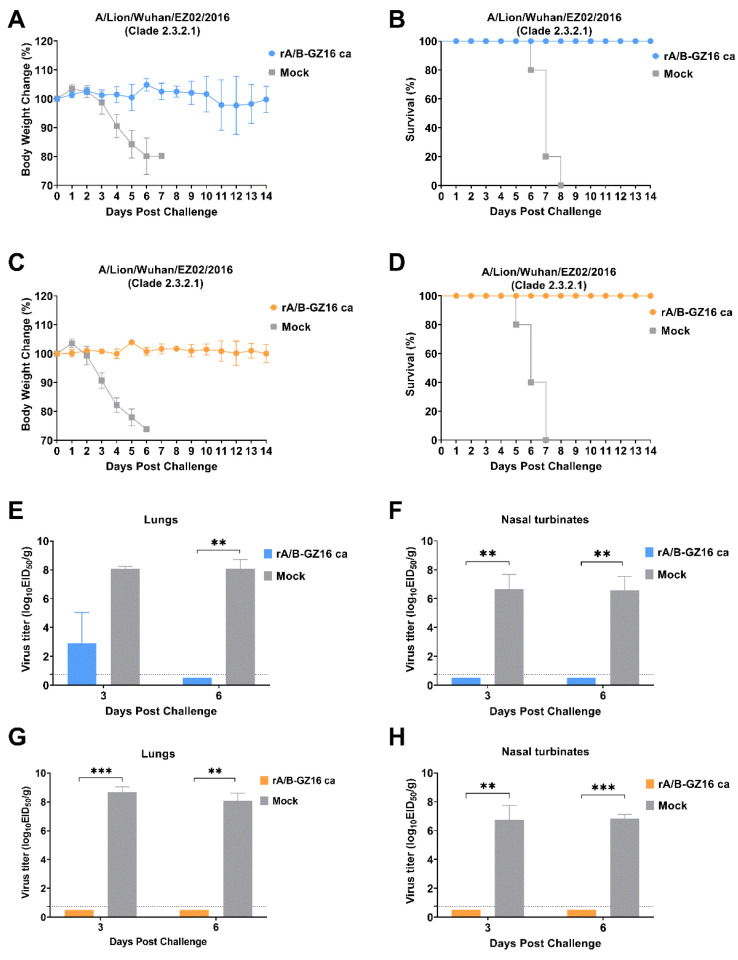
Vaccination with the H5 chimeric influenza vaccine protects BALB/c mice from lethal challenge with 10 MLD_50_ EZ02 at 50 μL. Body weight changes and survival were recorded for 14 days post-inoculation, *n* = 5. (**A**,**B**) One dose, (**C**,**D**) two doses. Virus replication in the lungs and nasal turbinates was measured on day 3 and day 6, *n* = 3. (**E**,**F**) One dose, (**G**,**H**) two doses. Viral titer is expressed as log_10_ EID_50_/g. The detection limit was set to 0.75 log_10_ EID_50_/g. Data are shown as the mean ± SD and were analyzed by an unpaired t-test or Mann-Whitney test. ** *p* < 0.01, and *** *p* < 0.001.

**Figure 9:**
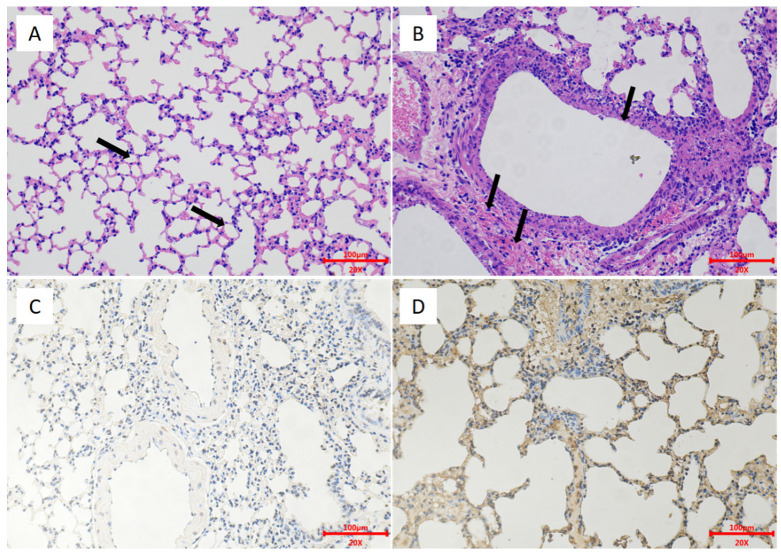
Lung HE and IHC of the two-dose group and the control group mice. The mice were euthanized on day 3 after the challenge. Images were captured at a 20× magnification. (**A**) Two-dose group mice, HE. (**B**) Control group mice, HE. (**C**) Two-dose group, IHC. (**D**) Control group, IHC.

To evaluate the protection afforded by the H5 vaccine, we challenged mice pre-immunized with one or two doses of the vaccine with the H5 wild-type virus EZ02. In the one-dose group, mice developed a small weight loss, but none died during the 14 days. The nonimmunized control group mice experienced severe weight loss and died at days 6–8 post-inoculation (Figure 8A,B). On days 3 and 6, the control group mice had high virus titers in lungs and nasal turbinates. The lung viral titer of the control group was 8.08 ± 0.14 log_10_ EID_50_/g at 3 dpi and 8.08 ± 0.63 log_10_ EID_50_/g at 6 dpi (Figure 8E). The nasal turbinate viral titer of the control group was 6.67 ± 1.01 log_10_ EID_50_/g at 3 dpi and 6.58 ± 0.95 log_10_ EID_50_/g at 6 dpi (Figure 8F). Conversely, the mice in the one-dose group revealed only low virus replication in the lungs on day 3 with the titers of 3.75 log_10_ EID_50_/g and 4.5 log_10_ EID_50_/g (Figure 8E). On day 6, no virus replication was recorded in the lungs and nasal turbinates of the one-dose group (Figure 8F). In the two-dose group, the nonimmunized control group mice experienced severe weight loss and died at day 5 to day 7 post-inoculation (Figure 8C,D). No virus replication was detected on days 3 and 6 in the lungs and nasal turbinates for the two-dose group (Figure 8G,H). The control group could show high virus titration in the lungs and nasal turbinates. The lung viral titer of the control group was 8.67 ± 0.38 log_10_ EID_50_/g at 3 dpi and 8.08 ± 0.52 log_10_ EID_50_/g at 6 dpi (Figure 8G). The nasal turbinate viral titer of the control group was 6.75 ± 1.00 log_10_ EID_50_/g at 3 dpi and 6.83 ± 0.29 log_10_ EID_50_/g at 6 dpi (Figure 8H). The HE and IHC results revealed pathological lesions in the lungs of the control group mice. There was bronchial epithelial erosion, interstitial inflammatory cell infiltration, and hyperemia (Figure 9). The HE and IHC results indicated no obvious lesions in the lungs, uniform alveolar arrangement, and standard alveolar septa in the two-dose group. These experiments suggested that two doses of the H5 chimeric candidate vaccine provided 100% protection against an HPAI H5 wild-type virus in the mice.

## 4. Discussion

The influenza virus contains A, B, C, and D types. Influenza A virus has the most subtypes and poses the greatest threat to human health, and has caused some pandemics in the past [36]. Influenza B virus can infect humans, but it only causes small outbreaks. The circulation of influenza B virus has not been as widespread as influenza A virus. The H5N1 avian influenza virus has been a threat to humans [37]. H5N1 causes huge agricultural and economic losses. Although human-to-human transmission of H5N1 is limited [38,39], we cannot neglect its potential threat. Fatality from this virus has been nearly 50%. Based on the WHO reports, from 2003 to 2009, the WHO confirmed 468 cases of H5N1 in humans, of which 282 died. From 2010 to 2014, there were 233 human cases and 125 deaths, while there were 160 human cases and 48 deaths in 2015–2019. We analyzed the H5 clades from all human cases of H5N1 infection from the Global Initiative of Sharing All Influenza Data (GISAID) website until August 2021. Among the human infections, most strains of H5N1 belonged to clades 0, 1, 2.1, 2.2, 2.3, 3, and 7. Egypt, Cambodia, China, Vietnam, and Indonesia have each reported a greater number of human H5N1 cases than any other country [9,10]. The H5N1 influenza vaccine needs to be studied for augmenting prevention and control.

Several prepared H5 candidate vaccines are underway in clinical trials. The candidates include inactivated H5 vaccines, recombinant HA-based vaccine, spilt virus or subunit vaccine, plant-derived influenza vaccine, insect-cell-derived virus-like particle, and live influenza vaccine [40,41,42]. Among the H5 pandemic vaccines, the live influenza vaccine has been studied by various master donors of influenza A and influenza B viruses. Live influenza vaccines provide the most cross-protection immune responses [43]. Recently, live influenza vaccines from different master donors were cold-adapted. This live influenza vaccine has been used in the USA, Russia, and Europe, but a live vaccine has the possibility of adaptation to grow in high temperatures in laboratory conditions. As humans are infected with different types of influenza A viruses, reassortment may occur in humans. However, reassortment between IAV and IBV has not been observed so far. In this study, we have designed a new chimeric influenza A/B strategy to overcome this problem.

Investigators have made chimeras between IAV and IBV viruses. Some past studies exchanged HA and NA genes while retaining the packaging signals [27]. However, they chose an influenza A backbone and placed HA and NA functional domains of IBV in backbones. We employed this chimeric strategy with a cold-adapted master donor of IBV and created a novel live influenza virus vaccine. Theoretically, this new vaccine can overcome the shortcomings of LAIV. In our experiments, we optimized the protocols for rescue, cell culture, and volume of plasmids. Our H5 chimeric virus vaccine can offer a new strategy for a live influenza vaccine.

Using reverse genetics technology, investigators have constructed a variety of recombined pathogens such as viruses and bacterial, especially type A influenza virus [19]. Based on various cloning methods, many chimeric vaccines have been studied. The master donors of influenza B virus, B/Leningrad/14/15, B/Victoria/2/87, B/Lee/40, B/Brisbane/60/2008, and other master donors, have been used so far [44,45,46,47,48]. Some live influenza B virus vaccines have been reported recently, but these study designs were based on different strategies. Most of the live influenza B virus vaccines chose NS1 or M2 genes to obtain the attenuated function [22,49]. However, all of these studies selected a wild-type influenza B virus. The influenza virus may grow poorly when we change the NS1 or other genes. Hence, we selected a cold-adapted type B influenza vaccine as a vaccine vector. Our vaccines can grow well in cells or eggs. Although there have been reports on the reassortment among influenza B viruses [50], the influenza B virus had a lower morbidity in humans. This vaccine vector is safe and attenuated in mice, and hence can be used against other pathogens. This new design has lots of advantages over other vaccines. The licensed live-attenuated cold-adapted influenza vaccine has been used in several countries [51].

We designed and constructed an H5N1 cold-adapted chimeric influenza virus vaccine through reverse genetics. For this purpose, we selected a virus candidate recommended by the WHO. Through a chimeric strategy, the modified HA gene could not recombine with the H5 circulating virus in nature. We evaluated the safety, immunogenicity, and protective efficacy by using a mice model. The results showed that our vaccine could induce a moderate amount of HAI, MN, and IgG antibodies. In addition, IgA antibodies were induced in the lung lavage fluids. The IgA antibodies indicated that the mucosal immunity had a pan protection. The cytokines secreted by cultured splenocytes increased in the immunized mice. IFN-γ and IL-4 were induced by the spleen lymphocytes and indicated cell-medicated immunity. Some reports suggested cell-mediated immunity in the tissues from the live influenza vaccine. Although we did not detect the CD4 and CD8 markers in the lungs, they were possibly detected in the tissue-resident memory T-cells. The memory B-cells and T-cells could play a vital function in inducing the influenza vaccine immunity [52,53,54]. The cell-mediated immune responses could provide cross-reactive T-cell responses.

The IgA antibodies were intranasally induced, which offered the advantage of the live influenza vaccine relative to vaccines based on inactivated influenza [55]. The mucosal immune response is well-developed in children and provides protection [12]. The IgA from the respiratory tract has been reported to protect naive mice, and IgA could elevate cross-reactivity [56]. We detected a high amount of IgA antibodies in the mouse lavage fluids. Other animal models, such as ferret or nonhuman primate models, have been employed to evaluate influenza vaccines. This H5 influenza vaccine may continue to be tested in future studies. The nasal fluid and the lung lavage fluids can be collected and determined to detect IgA antibody responses.

In terms of the protective efficacy of immunized mice, we selected a wild-type H5N1 influenza virus for the challenge. We found that two doses of our H5 influenza vaccine provided 100% protection from wild-type viruses. We did not detect any virus replication in the lungs and nasal turbinates. In the one-dose group, we recorded protection, although we detected the virus in the lungs at day 3 post-immunization. Thus, a two-dose inoculation seemed more effective. The protein microarray assay found that the immunized mice serum demonstrated a greater cross-reactivity for different clades of H5 HA proteins. These results suggest that the H5N1 cold-adapted chimeric influenza vaccine offers greater protection against other clade H5 wild-type viruses.

In summary, we designed an H5N1 chimeric influenza virus vaccine based on a cold-adapted master donor of the influenza B virus. This H5N1 chimeric vaccine offers the advantages of ease of administration, induction of IgA antibodies, and cellular immunity. The proposed H5N1 vaccine has potential as a pandemic-preparedness vaccine.

## Figures and Tables

**Table 1:** Results of temperature-sensitive (ts) and cold-adapted (ca) assays of the H5 candidate vaccine.

Virus	Virus Titer [log_10_(EID_50_ mL^−1^)]			RCT_39_	RCT_27_	Phenotype
	33 °C	39 °C	27 °C			
**rA/B-GZ16 ca**	8.2 ± 0.2	-	6.9 ± 0.7	8.2	1.3	ts, ca
**BV99**	7.7 ± 0.5	-	6.2 ± 0.2	7.7	1.5	ts, ca

## Data Availability

The gene sequences used in this study are openly available in GenBank and GISAID.

## Supplementary Materials

The following are available online at https://www.mdpi.com/article/10.3390/v13122420/s1, Figure S1: All H5 HA proteins listed in the protein microarrays, Table S1: The PCR amplification primers for IBV full genomes.
