# Supplementary material for: Safety, Immunogenicity, and Protective Efficacy of an H5N1 Chimeric Cold-Adapted Attenuated Virus Vaccine in a Mouse Model
Source: Viruses. 2021 Dec 3;13(12):2420. doi: 10.3390/v13122420 (PMC8709164; doi:10.3390/v13122420)
Supplement: Supplementary file 1 [file viruses-13-02420-s001.zip › viruses-1477289-supplementary.pdf]

Table S1. The PCR amplification primers for IBV full genomes.

Amplified fragments	Forward Primer（5′-3′）	Reverse Primer（5′-3′）
PB2	CCAGCAGAAGCGGAGCGTTT	TTATGAGAAACACGAGCATTTTTCACTC
PB1	CCAGCAGAAGCGGAGCCTTT	TTAGTAGAAACACGAGCCTTTTTTCA
PA	CCAGCAGAAGCGGTGCGTTT	TTAGTAGAAACACGTGCATTTTTAATTC
HA	CCAGCAGAAGCAGAGCATTTTC	TTAGTAGTAACAAGAGCATTTTTCAATAACG
NP	CCAGCAGAAGCACAGCA	TTAGTAGAAACAACAGCATTTTT
NA	CCAGCAGAAGCAGAGCATC	TTAGTAGTAACAAGAGCATTTTTCAG
M	CCAGCAGAAGCACGCACT	TTAGTAGAAACAACGCACTTTTTC
NS	CCAGCAGAAGCAGAGCA	TTAGTAGTAACAAGAGGATTTTTAT

Source	Catalog No.	Strain Name	Subtype	Clade	Molecule[a]	Express System	Tags	GenBank/GISAID[b]
Sino Biological	40024-V08B	A/Goose/Guangdong/1/96	H5N1	0	HA1+ HA2	Baculovirus	His	YP_308669.1
Sino Biological	11689-V08H	A/Hong Kong/483/97	H5N1	0	HA1+ HA2	Human cells	His	AAC32099.1
Sino Biological	11713-V08H	A/Hong kong/213/2003	H5N1	1	HA1+ HA2	Human cells	His	ABP51975.1
Sino Biological	10003-V06H3	A/VietNam/1203/ 2004	H5N1	1	HA1+ HA2	Human cells	His+ Fc (mouse)	AAW80717.1
Sino Biological	11710-V08B	A/Cambodia/R0405050/2007	H5N1	1.1	HA1+HA2	Baculovirus	His	n.s.
Sino Biological	40026-V08H	A/Cambodia/S12 11394/2008	H5N1	1.1	HA1+ HA2	Human cells	His	ADM95445.1
Sino Biological	11698-V08H	A/duck/Hunan/79 5/2002	H5N1	2.1.1	HA1+ HA2	Human cells	His	ACA47835.1
Sino Biological	11060-V08H2	A/Indonesia/5/20 05	H5N1	2.1.3.2	HA1+ HA2	Human cells	His	ABW06108.1
Sino Biological	40004-V08H	A/Xinjiang/1/2006	H5N1	2.2	HA1+ HA2	Human cells	His	ACJ68614.1
Sino Biological	40117-V08B	A/bar-headed goose/Qinghai/1A /2005	H5N1	2.2	HA1+ HA2	Baculovirus	His	ABF93441.1
Sino Biological	11697-V08H	A/Egypt/2321- NAMRU3/2007	H5N1	2.2.1	HA1+ HA2	Human cells	His	ABP96850.1
Cambridge Biologics	01-02-0546	A/Egypt/N03072/2010	H5N1	2.2.1	HA1	Human cells	His	EPI255379
Sino Biological	40049-V08H1	A/Egypt/3300- NAMRU3/2008	H5N1	2.2.1.1	HA1	Human cells	His	ACI06185.1
Sino Biological	11048-V08H1	A/Anhui/1/2005	H5N1	2.3.4	HA1+ HA2	Human cells	His	ABD28180.1
Sino Biological	11694-V08H	A/Japanese white-eye/Hong Kong/1038/2006	H5N1	2.3.4	HA1+ HA2	Human cells	His	ABJ96775.1
Cambridge Biologics	01-02-0555	A/chicken/Bangladesh/11rs1984-30/2011	H5N1	2.3.4.2	HA1	Human cells	His	AEQ50043
Cambridge Biologics	01-02-0570	A/Guizhou/1/2013	H5N1	2.3.4.2	HA1	Human cells	His	n.s.
Sino Biological	40022-V08H1	A/Vietnam/UT314 13II/2008	H5N1	2.3.4.3	HA1	Human cells	His	ADF83651.1
Sino Biological	40088-V08H1	A/chicken/Yamag uchi/7/2004	H5N1	2.5	HA1	Human cells	His	BAD89305.1
Sino Biological	11690-V08H	A/goose/Guiyang/ 337/2006	H5N1	4	HA1+ HA2	Human cells	His	ABJ96698.1
Sino Biological	40372-V08B	A/chicken/Jilin/9/ 2004	H5N1	5 (1-2-5-6-8-9-like)	HA1+ HA2	Baculovirus	His	AAT76166.1
Sino Biological	40158-V08B2	A/chicken/VietNam/NCVD-016/2008	H5N1	7.1	HA1+ HA2	Baculovirus	His	ACO07033.1
Cambridge Biologics	01-02-0542	A/chicken/Viet Nam/NCVD-03/2008	H5N1	7.1	HA1	Human cells	His	n.s.
Sino Biological	40015-V08B	A/Hubei/1/2010	H5N1	2.3.2.1a	HA1+ HA2	Baculovirus	His	AEO89181.1
Sino Biological	40160-V08B1	A/barnswallow/H ongKong/D10-1161/2010	H5N1	2.3.2.1b	HA1+ HA2	Baculovirus	His	AGC13463.1
Sino Biological	40044-V08H	A/common magpie/Hong Kong/5052/2007	H5N1	2.3.2.1	HA1+ HA2	Human cells	His	ACJ26242.1
Sino Biological	MF15JU2412	A/Chicken/Guizhou/1153/2016	H5N1	2.3.2.1d	HA1+ HA2	Baculovirus	His	MT126478.1
Cambridge Biologics	01-02-0575	A/chicken/Ghana/20/2015	H5N1	2.3.2.1f	HA1	Human cells	His	AMX74637
Sino Biological	40495-V08B	A/Sichuan/26221/2014	H5N6	2.3.4.4a	HA1+ HA2	Baculovirus	His	EPI533583
Cambridge Biologics	01-02-0572	A/tufted duck/Germany-SH/R8444/2016	H5N8	2.3.4.4b	HA1	Human cells	His	EPI860509
Sino Biological	MF15JL0521	A/Fujian-Sanyuan/21099/2017	H5N6	2.3.4.4b	HA1+ HA2	Baculovirus	His	EPI1202729
Cambridge Biologics	01-02-0566	A/Cygnus atratus/Hubei/2Z2-O/2016	H5N8	2.3.4.4b	HA1	Human cells	His	ART29846
Cambridge Biologics	01-02-0569	A/northern pintail/Washington/40964/2014	H5N2	2.3.4.4c	HA1	Human cells	His	n.s.
Cambridge Biologics	01-02-0571	A/chicken/Iowa/04-20/2015	H5N2	2.3.4.4c	HA1	Human cells	His	AKG52687
Cambridge Biologics	01-02-0573	A/Hubei/29578/2016	H5N6	2.3.4.4d	HA1	Human cells	His	n.s.
Cambridge Biologics	01-02-0565	A/chicken/Hubei/ZYSJF11/2016	H5N6	2.3.4.4d	HA1	Human cells	His	n.s.
Cambridge Biologics	01-02-0574	A/duck/Hyogo/1/2016	H5N6	2.3.4.4e	HA1	Human cells	His	n.s.
Sino Biological	40465-V08B	A/duck/Guangdong/GD01/2014	H5N6	2.3.4.4f	HA1+ HA2	Baculovirus	His	AIA24403.1
Sino Biological	MF15JL0221	A/Guangdong/18SF020/2018	H5N6	2.3.4.4h	HA1+ HA2	Baculovirus	His	EPI1352813

a HA1, hemagglutinin head; HA2, hemagglutinin stem;
b n.s., not supplied by company.

Figure S1. All H5 HA proteins listed in the protein microarrays. The purified HA proteins were obtained from Sino Biological and Cambridgebio Biologics. These HA proteins belongs to different clades of H5 subtype influenza virus. The figure shows the virus strain, subtype, GenBank/GISAID number, clade or subclade, cell expression system, and catalog number for each HA protein..
